# HT-29 Colon Cancer Cell Electromanipulation and Assessment Based on Their Electrical Properties

**DOI:** 10.3390/mi13111833

**Published:** 2022-10-27

**Authors:** Marius Andrei Olariu, Catalin Tucureanu, Tudor Alexandru Filip, Iuliana Caras, Aurora Salageanu, Valentin Vasile, Marioara Avram, Bianca Tincu, Ina Turcan

**Affiliations:** 1 Department of Electrical Measurements and Materials, Faculty of Electrical Engineering and Information Technology, Gheorghe Asachi Technical University of Iasi, 700050 Iasi, Romania; 2 Cantacuzino, National Medical-Military Institute for Research and Development, 050096 Bucharest, Romania; 3 Academy of Romanian Scientists, Splaiul Independentei 54, 050094 Bucharest, Romania; 4 National Institute for Research and Development in Microtechnologies-IMT Bucharest, 077190 Bucharest, Romania; 5 DDS Diagnostic SRL, 7 Vulcan Judetu Street, 030423 Bucharest, Romania; 6 Faculty of Applied Chemistry and Material Science, University Politehnica of Bucharest, 060042 Bucharest, Romania

**Keywords:** cancer cells, dielectrophoresis, electrical impedance spectroscopy

## Abstract

This study proposes a feasible approach for the rapid, sensitive, and label-free identification of cancerous cells based on dielectrophoretic (DEP) manipulation and electrical characterization. In this method, the concentration of target cells at the level of customized microelectrodes via DEP is first determined, followed by an electrical impedance evaluation. The study demonstrates the capacity of the methodology to electrically differentiate HT-29 cancer cells from healthy blood cells based on their impedance spectra. Within a higher frequency domain, the electrical impedance of trapped cancer cells was significantly lower compared with the normal ones. In order to evaluate the functionality and reproducibility of the proposed method, the influence of the DEP and EIS (electrical impedance spectroscopy) operating voltages on the electrical characterization of trapped HT-29 cells was analyzed.

## 1. Introduction

According to the American Cancer Society, colorectal cancer is among the most frequently diagnosed cancers in the United States. For 2022, it is estimated that about 106,000 persons (both men and women) will be diagnosed with colorectal cancer while the number of deaths caused by this type of cancer will reach about 52,000 [1]. Moreover, at the level of EU-27, according to the European Cancer Information System, in 2020, colorectal cancer accounted for about 340,000 new cases, about 12.7% of all new cancer cases diagnosed, and about 12.4% of all the deaths caused by cancer [2]. Considering this worrying data, the necessity of developing new tools for the rapid detection of colorectal cancer is more than justified and alternative solutions for ensuring smooth and easily usable methods should be studied, developed and released on the market as soon as possible.

Among the emerging methods proposed by researchers for the rapid and cost-effective detection of cancer, dielectrophoresis (DEP) is one of the top technologies. Dielectrophoresis (DEP) is the electrokinetic motion of dielectrically polarizable particles in a fluid under the action of non-uniform electric fields. DEP has been successfully employed for the identification of various types of cancerous cells, including breast cancer (MCF-7, MDA-MB-435, MDA-MB-468, and MDA-MB-231 cell lines), ovarian cancer (SKOV-3), prostate cancer (LnCap, PC3 and RWPE-1 cell lines) and leukemia (K526 and Jurkat cells) [3,4,5].

Colorectal cancer detection has also been explored, with plenty of studies being published in recent decades. Fang Yang et al. employed conventional dielectrophoresis in a microfluidic chip to manipulate and isolate colorectal cancer HCT-116 cells from a mixture with human embryonic kidney 293 (HEK 293) and E. coli cells [6]. In [7], a DEP method based on the capture voltage spectrum was proposed for measuring the dielectric properties of HT-29 cells and separating these cells from other ones (such as red blood cells). Alshareef et al. have demonstrated the ability of a contactless, label free, highly specific, DEP-based microfluidic separation system to separate cancerous epithelial cells of one type (MCF-7) from another similar cell type (HCT-116) [8]. Additionally, the performance of the proposed DEP sorter was analyzed by measuring the relationship between the enrichment factor and operational parameters, such as AC frequency, voltage, and flow rate, attaining enrichment efficiencies of as high as 93% (0.1 μL/min, 9 Vpp, 3.2 MHz).

Recently, in a combined electrical approach, DEP and IA (impedance analysis) were implemented by Velasco et al. in a novel lab-on-a-chip platform capable of screening (real-time and continuous) drug efficacy on (cancer) cell subpopulations without the need for a fluorescent/magnetic label, optical system, or large sample/reagent volume [9]. The results demonstrated the capability of the platform to measure and monitor the cancer (HCT-116) cells’ response to a therapeutic agent (SN38) in real-time, while simultaneously validating the effectiveness of a therapeutic agent on cells of interest. Moreover, by equipping DEP with additional technologies which can determine a cell’s “electrical signature”, such as electrical impedance spectroscopy (EIS), the identification of different malignant cells can be facilitated [10,11,12,13]. In spite of the fact that both DEP and EIS were separately or complementarily employed in studies aiming to sort, isolate and characterize biological cells based on a dielectric properties analysis, neither of these two techniques have been widely accepted in pre-clinical and clinical practice due to a lack of standardization of the practical procedures. However, in order to foster a wide-scale acceptance of these techniques, an increase in the number of studies envisaging DEP and EIS employability on biological cells analysis is needed and experimental results should be validated at first at the laboratory level. Moreover, plenty of studies were published regarding the characterization of cancerous cells via EIS but the majority of these studies are based on an evaluation of cells suspended within a liquid [3]. In our opinion, these kinds of studies do not provide sufficient information on the biophysical state of the cells but rather on the macroscopic dielectric state of the suspension medium.

To the best of our knowledge, a previous study envisaging the dielectric analysis of trapped HT-29 cells is not available within the scientific literature. Within this study, we present an experimental study for the identification of colon cancer cells, HT-29, from normal ones by employing a practical laboratory method which uses a combination of DEP and EIS. The method first proposes the trapping of target biological cells at the level of an array of interdigitated microelectrodes; this is followed by a second step which supports the determination of trapped cells’ dielectric characteristics [13]. The derivation of the basic principle of the method, as well as the operating parameters that demonstrate its feasibility, are discussed herein, along with the impedance differentiation of HT-29 cancer cells from healthy blood cells.

## 2. Materials and Methods

### 2.1. Cell Culture and Sample Preparation

#### 2.1.1. Cell Lines

Human colon adenocarcinoma cell line HT-29 (Cat. No. 91072201) and human monocyte-like THP-1 cells (Cat. No. 88081201) were purchased from the European Collection of Authenticated Cell Cultures (ECACC) and cultured in McCoy’s 5A Modified Medium (Bio Whittaker Lonza, Verviers, Belgium) and RPMI-1640 (Bio Whittaker Lonza, Verviers, Belgium) respectively; they were supplemented with 10% fetal bovine serum (FBS, Euroclone, Milan, Italy) and 100 IU/mL penicillin + 100 μg/mL streptomycin (Lonza, Basel, Switzerland) (complete culture medium). Cell lines were incubated at 37 °C in an atmosphere supplemented with 5% CO_2_, in 75 cm^2^ flasks. The adherent cell line, HT-29, was cultured until ~85% confluence; it was then washed with phosphate-buffered saline (PBS, Merck, Darmstadt, Germany), and detached using 0.05% trypsin-EDTA solution (Thermo Fischer Scientific, Waltham, MA, USA). The cells were then suspended in a complete culture medium, washed by centrifugation at 200× *g* for 10 min, and then re-suspended in a fresh complete culture medium. The non-adherent cell lines (THP-1) were simply collected and centrifuged in the previously mentioned conditions.

#### 2.1.2. Human Peripheral Blood Mononuclear Cells

Human blood was obtained from a healthy donor (lab worker) after obtaining informed consent and ethical approval. Peripheral blood mononuclear cells (PBMCs) were isolated by using Ficoll-Hypaque (1.077 g/mL density, (Merck, Darmstadt, Germany)) and re-suspended in RPMI medium.

#### 2.1.3. Suspension Medium

The low conductivity suspension medium (250 mM sucrose, 13 mS/m conductivity) was chosen based on viability data and from an electrical viewpoint in preliminary experiments. In order to maintain osmotic pressure, 250 mM sucrose (Merck, Darmstadt, Germany) dissolved in distilled water acted as the base solution with a conductivity of 0.5 mS/m. In order to increase conductivity and adjust the pH to 7.4, a 250 mM HEPES (Merck, Darmstadt, Germany) solution was used. The osmolarity was measured with a VAPRO (Vapor Pressure Osmometer Model 5600) and was 250 mmol/kg. The conductivity measurement was performed with a ZetaSizer Nano-2S.

#### 2.1.4. Sample Preparation and Viability Assay

Cancer cell lines and normal PBMC were washed and re-suspended in a low conductivity suspension medium (2 × 10^6^ cells/mL). Their viability was evaluated before and after DEP measurements by staining the cells with Acridine Orange/Propidium Iodide Stain stock solution. After incubation at 37 °C 5% CO_2_ for 24 h, the standard medium was replaced with the low conductivity solution, and a PBS solution was used for comparison. Using a script, a total of nine images per well were obtained using a Nikon Eclipse Ti (Nikon, Minato City, Tokyo, Japan) inverted microscope equipped with a Nikon DS-Qi2 monochrome camera (Nikon, Minato City, Tokyo, Japan). The images were processed using ImageJ software. Cell viability was comparable with the cells in PBS and was over 95% after 24 h (data not included).

### 2.2. Experimental Set-Up and Equipment

The experimental activity in this study involved two major steps: (1) trapping the cells at the microelectrode level via DEP and (2) identification of the cell’s type by measuring its impedance characteristics, as presented in our previous study [13]. In order to ensure the development of higher gradient field regions, an interdigitated microelectrode with a castellated architecture was selected. The interdigitated microelectrode array had 16 fingers with a length of 2560 µm; the gap between the fingers and the intercalations had a dimension of 40 µm. A Keysight 33521A Function/Arbitrary Waveform Generator was employed to generate a sinusoidal AC electric field. The cells’ distribution and motion at the microelectrode level was monitored and recorded using an improvised optical setup consisting of a Nikon Plan Fluor 10 ×/0.30 microscope with a mounted CCD Nikon Digital Sight DS-Qi1Mc camera connected to a computer that was running NIS-Elements AR 3.0 SP 1 (Build 455) software. All image processing was performed in Fiji/ImageJ [14]. Individual microscopy frames were drift corrected to reduce residual displacement. The temporal averaged image for each acquisition was subtracted to reduce the contribution of light scattered by the electrode. Temporal stacks were further Fourier filtered with cut-offs of 5 to 25 μm, corresponding to the dimensions of individual cells, to reduce repetitive patterns from the electrode. Cell velocities under the DEP field were calculated using TrackMate [15] in ImageJ, starting with Fourier filtered images. Individual cells were identified with a find maxima filter and only cells with sufficient detection quality across frames were tracked (a minimum of 500 cells/condition). Electrical impedance spectroscopy measurements were performed using a Novocontrol Broadband Dielectric Spectrometer (Alpha-A High-Performance Frequency Analyzer).

## 3. Results and Discussions

Human colon cancer cell line HT-29, human monocyte-like THP-1 cells, and human peripheral blood mononuclear cells were used in experiments to evaluate the performance of the microchip. A solution containing a specific number (2 × 10^6^ cells/mL) of cells was dropped onto the microelectrode sensing area (5 μL). The effect of the EIS and DEP operating voltages from the viewpoint of impedance characterization and cell viability was evaluated. In order to generate a p-DEP force in our studies, four different sinusoidal excitation voltages (3, 6, 9 and 12 Vpp) of a fixed frequency of 1 MHz were applied to the electrodes, for about 5 min, providing sufficient time to concentrate the cells. Each time, before and after cell trapping, the impedance-based measurement was performed.

**Figure 1:**
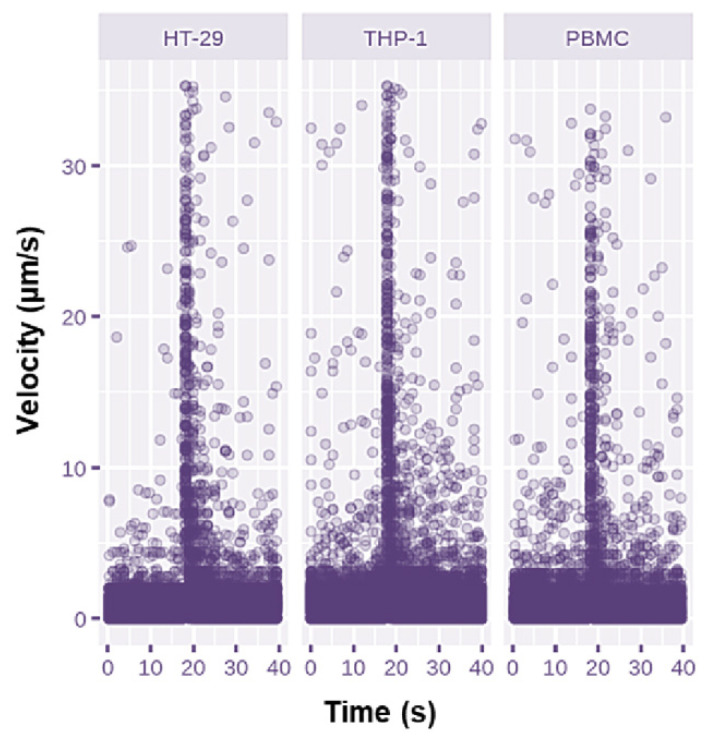
Cell velocities distribution measured with the applied voltage of Vpp = 12 V at 1 MHz. Cells were allowed to settle for 15 s in each acquisition in order to measure baseline displacement before the application of the DEP field.

Under the effect of p-DEP, all the cells began to move towards the castellation of interdigitated fingers, immediately after the AC electric field was applied, and after a few seconds they were concentrated at the electrode surface for all the applied fields (see the Supplementary Materials). Cell velocities at the moment of DEP application were calculated starting from the distance of cell movement and the time needed to go through it. Figure 1 depicts cell velocities measured under the action of an AC voltage of Vpp = 12 V. The HT-29 and THP-1 cells moved towards the electrode edges with a higher speed compared to normal cells due to their larger sizes and the cubic relation of the DEP force to the cell radius.

**Figure 2:**
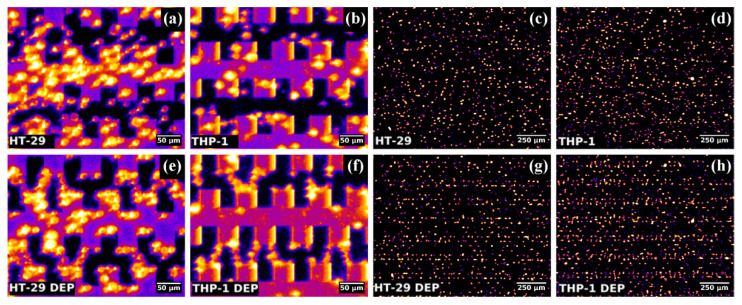
Microscopy images of a cell sample’s (HT-29 and THP-1) distribution before (**a**–**d**) and after (**e**,**f**) DEP manipulation: (**a**,**b**,**e**,**f**) zoomed in pseudocolored raw images of oblique illuminated cells showing “cell bridges” along electric field lines after DEP; (**c**,**d**,**g**,**h**) Fourier filtered images covering the full microscopic field used for particle tracking showing organized deposition of cells on the electrode after DEP.

The microscopic images of cell samples before and after (5 min) 12 Vpp application of DEP are depicted in Figure 2; it can be noticed that the majority of the cells were attracted, under DEP force, to the tips/castellations of the microelectrodes, i.e., towards the highest electric field regions. Moreover, in some regions, the cells followed electric field lines between adjacent microelectrodes, due to their high interfacial polarization, creating “cells’ bridges”.

**Figure 3:**
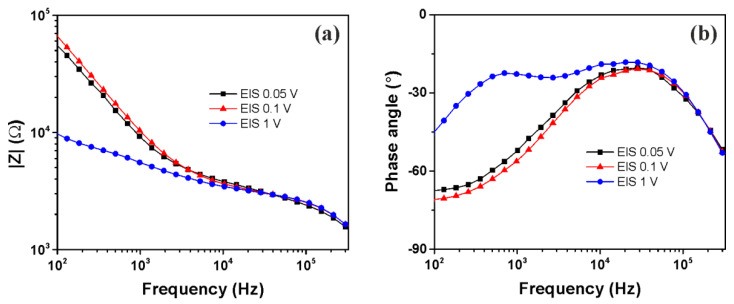
Impedance magnitude (**a**) and phase angle (**b**) of trapped HT-29 cells at various EIS operating voltages.

In order to evaluate the influence of EIS operating voltages on the electrical characterization of trapped HT-29 cells (6 Vpp, 1 MHz), impedance measurements were performed within the frequency range from 0.1 to 300 kHz at 0.05, 0.1 and 1 V (Figure 3). The transition from capacitive behavior, which dominates at lower frequencies, to resistive behavior that prevails at higher frequencies was highlighted. At low frequencies, increasing the EIS operating voltage to 1 V led to a significant decrease in the magnitude of cells’ impedance accompanied with an interesting different feature of the phase angle at below 10 kHz. In contrast, at high frequencies >10^4^ Hz, the impedances of HT-29 cells are equal regardless of operating voltage. The behavior of impedance spectra at an operating voltage of 1V can be explained in terms of crossover frequency.

The DEP crossover frequency is the characteristic frequency at which the polarity of the dielectrophoretic force changes. After this frequency value, the cells exhibit a negative dielectrophoretic force. The crossover frequency of HT-29 cancer cells was determined by observing the motion of cells at the electrode edges when the frequency applied was slowly changed to around 34 kHz. Therefore, during impedance measurements at 1V, as the frequency decreases, a disturbance of the cells occurs while approaching crossover frequency, even for such a low voltage. This explains the fact that, at low frequencies, the impedance values are higher in the case of 0.05 and 0.1 V as most of the current is forced to flow in the solution channels under and between adherent cells; this is in contrast to 1 V, for which, at 34 kHz, the cells were rejected (not all) from the electrode surface. Moreover, in the case of a 1V voltage, the double layer capacitance phenomenon is more pronounced, a fact which led to a decrease in impedance at low frequencies. Therefore, it is essential to use a low EIS operating voltage in order to not disturb the trapped cells and thus not affect the cells’ impedimetric characterization. Considering these aspects, the EIS operating voltage of 0.1 V was chosen for performing the following experiments.

**Figure 4:**
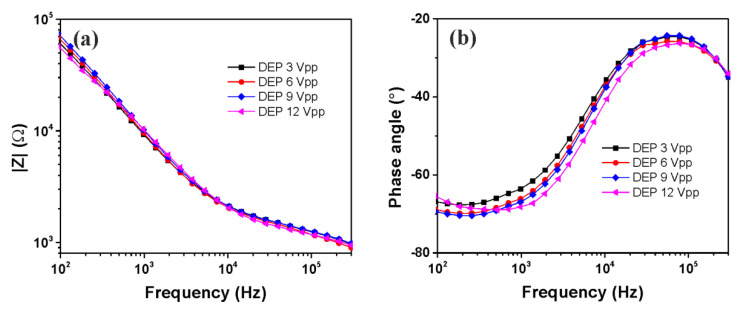
Impedance magnitude (**a**) and phase angle (**b**) of trapped HT-29 cells at different DEP operating voltages.

The functionality and reproducibility of the proposed method was evaluated, starting from the EIS responses of trapped cells at different DEP operating voltages (3, 6, 9, and 12 Vpp) with four independent interdigitated microelectrodes that were fabricated according to a similar procedure. Based on the impedance magnitude and phase angle frequency dependencies illustrated in Figure 4, no significant differences in the EIS responses of HT-29 were observed, even if the DEP voltage was changed. Table 1 depicts the average values of the impedance magnitude and phase angle, the standard deviation (SD), and the relative standard deviation (RSD) that were calculated between the electrodes at three different frequencies (10^3^, 10^4^, and 10^5^ Hz). The reproducibility of the EIS experiments realized following DEP cells’ trapping was found to be good with a % RSD of less than 5% in all investigated frequency ranges.

**Figure 5:**
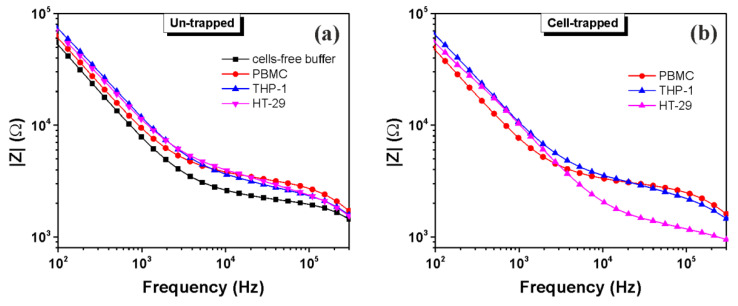
Impedance magnitude of different cell types (PBMC, THP-1 and HT-29) suspended in buffered sucrose solution, before (**a**) and after cells trapping (DEP: 12 Vpp at 1MHz for about 5 min) (**b**).

In order to differentiate HT-29 from normal cells (PBMC and THP-1), the impedance measurements at a 0.1 V EIS voltage of the un-trapped and trapped living cells were performed (Figure 5). The impedance magnitude of the suspension medium (cell-free buffer) decreases with increasing frequency, representing a transition from a capacitive to resistive behavior as the applied signal crosses from low to high (Figure 5a). Since the media is more conductive than the suspended cell samples, the impedance of the media is lower. However, initially, before DEP trapping, when cells are suspended within the entire volume of the suspension solution, no significant differences were observed between the different cell types. In contrast, in the presence of DEP forces applied for 5 min, significant differences in the impedance spectra were observed among the various cell types (Figure 5b). As a consequence of the cells’ migration towards the electrode surface under p-DEP, as shown in Figure 2, the local ionic environment at the electrode/suspension medium interface is affected due to the highly insulating cell membrane. Because of their unique morphology and dielectric composition, each cell type provides individual impedance contributions.

At low frequencies, the current is forced to flow around the insulating cell membranes, while at higher frequencies, the current penetrates the cell membranes and flows through intracellular and extracellular fluid [16,17]. Therefore, differences noticed at frequencies of below 30 kHz between human peripheral blood mononuclear cells and cell lines (THP-1 and HT-29) could be attributed to the surface morphological features of the cell membranes and to the electrode surface area, which is covered with cells, i.e., cells radii [13]. The colon cancer cells (HT-29) exhibited a lower impedance magnitude (0.95–3.7 kΩ) than normal cells (PBMC and THP-1) in the frequency range from 3 to 300 kHz. Other impedance investigations on the human breast cancer cell lines (MCF-7, MCF-MB-231, and MDA-MB-435) [18], bladder cancer cell lines (grade III: T24 and grade II: TSGH8301) [19], cervical cancer cells [20] and human lung cancer cells (H358, A549) [21] of different pathological grades also indicated a similar tendency of cancer cells which possessed a lower impedance magnitude. The differences in impedance response are presumably caused by cellular factors such as physiological states, membrane properties, the ion concentration in the cytoplasm, and the cell size [21,22]. Thus, HT-29 and THP-1 cells have similar biophysical responses at low frequencies (Figure 5b) as both types of cells are, to a large extent, identical from a morphological viewpoint. The lower impedance magnitude of trapped HT-29 within a high frequency domain is higher in comparison to the cell-free suspension medium (Figure 5a) due to the fact that the conductivity is a parameter influenced by the internal characteristics of the cells, i.e., cytoplasm conductivity, which apparently is higher in comparison to that of normal cells.

The measured results of the electrical properties of the studied cell samples In suspension clearly depicted that the electrical signatures of the cancer cells were distinctly different from those of the normal ones.

## 4. Conclusions

Dielectrophoresis (DEP) and electrical impedance spectroscopy (EIS) have been jointly employed for the successful electromanipulation and electrical characterization of HT-29 colorectal cancer cells. The study emphasizes the capacity of the methodology for the electrical differentiation of HT-29 cancer cells from healthy blood cells suspended in a low-conductivity medium. The electrical characteristics of the cancerous cells are discussed in terms of dielectric parameters, such as impedance and phase angle. The good reproducibility of the method is discussed with respect to four different applied voltages while the calculated % RSD was less than 5% in the case of DEP operating voltages. With respect to the EIS measurements, the applied voltage should be carefully chosen and considered in order to avoid an eventual disturbance of the trapped cells which may affect the impedance spectra.

## Figures and Tables

**Table 1:** The average values of the impedance magnitude and phase angle, the standard deviation (SD), and the relative standard deviation (RSD) calculated between the electrodes at different frequencies.

	10^3^ (Hz)			10^4^ (Hz)			10^5^ (Hz)		
	Mean Value	SD	RSD (%)	Mean Value	SD	RSD (%)	Mean Value	SD	RSD (%)
**│Z│ (** **Ω** **)**	9859.9	427.3	4.33	2066.1	32.5	1.57	1196.1	35.9	3.00
**Phase angle (°)**	−66.2	1.7	2.53	−37.6	1.8	4.84	−25.9	0.6	2.49

## Data Availability

The data that support the findings of this study are available from the corresponding author (I.T.).

## Supplementary Materials

The following supporting information can be downloaded at: https://www.mdpi.com/article/10.3390/mi13111833/s1, Video S1: Demonstration of HT-29 cells entrapment at the microelectrode level via Dielectrophoresis (DEP), Video S2: Demonstration of HT-29 cells entrapment at the microelectrode level via DEP (Fourier filtered images).
